# Suitability of Root and Rhizome Anatomy for Taxonomic Classification and Reconstruction of Phylogenetic Relationships in the Tribes Cardueae and Cichorieae (Asteraceae)

**DOI:** 10.3390/scipharm84040585

**Published:** 2016-05-27

**Authors:** Elisabeth Ginko, Christoph Dobeš, Johannes Saukel

**Affiliations:** 1 Department of Pharmacognosy, Pharmacobotany, University of Vienna, Althanstrasse 14, Vienna A-1090, Austria; 2 Department of Forest Genetics, Research Centre for Forests, Seckendorff-Gudent-Weg 8, Vienna A-1131, Austria

**Keywords:** anatomy, asteraceae, character evolution, pharmacy, phylogeny

## Abstract

The value of root and rhizome anatomy for the taxonomic characterisation of 59 species classified into 34 genera and 12 subtribes from the Asteraceae tribes Cardueae and Cichorieae was assessed. In addition, the evolutionary history of anatomical characters was reconstructed using a nuclear ribosomal DNA sequence-based phylogeny of the Cichorieae. Taxa were selected with a focus on pharmaceutically relevant species. A binary decision tree was constructed and discriminant function analyses were performed to extract taxonomically relevant anatomical characters and to infer the separability of infratribal taxa, respectively. The binary decision tree distinguished 33 species and two subspecies, but only five of the genera (sampled for at least two species) by a unique combination of hierarchically arranged characters. Accessions were discriminated—except for one sample worthy of discussion—according to their subtribal affiliation in the discriminant function analyses (DFA). However, constantly expressed subtribe-specific characters were almost missing and even in combination, did not discriminate the subtribes. Most anatomical characters showed at least some degree of homoplasious evolution limiting their suitability as phylogenetically informative characters.

## 1. Introduction

The Asteraceae, the sunflower family, represent one of the largest plant families comprising more than 23,000 species and about 1600 genera [1]. Based on molecular phylogenetic reconstructions the family is currently divided into 12 subfamilies, three of which, the Asteroideae, Carduoideae, and Cichorioideae, occur in Europe [2,3]. These three subfamilies comprise a large number of representatives in Central Europe, where they are rich in pharmaceutically used species, several of which are treated in European pharmacopeias [4,5]. The identification of pharmaceutically used species in pharmacopeias traditionally relies, aside from phytochemical characterisation, on anatomy. Anatomical characteristics are thus mandatory for pharmaceutical identification and purity control of drugs. Nevertheless, detailed comparative studies of the root/rhizome anatomy of species of the Carduoideae including the tribe Cardueae and also the Cichorioideae including the tribe Cichorieae are rare [6,7,8,9].

The speciose Cardueae comprise more than 2360 species organized into 73 genera [10] accounting for more than 90% of the species diversity in the Carduoideae [2]. The tribe exhibits high morphological diversity and holds some of the most species-rich genera (e.g., *Centaurea, Saussurea*) of the Asteraceae. Traditionally, the Cardueae were divided into four subtribes (Echinopsinae, Carlininae, Carduinae, Centaureinae). However, this tribal classification has been controversially discussed in the past (e.g., [11,12]). Recently, a new classification of the Cardueae comprising five subtribes—the four subtribes mentioned above plus subtribe Cardopatiinae—was suggested [10]. Pharmaceutically important species are *Carlina acaulis* L. [13], *Arctium lappa* L., and *Arctium tomentosum* Mill. [14] to mention only a few.

The Cichorieae (Cichorioideae) comprise 93 genera [15]. Reported species numbers differ widely, depending on the taxonomic concept applied to classify the numerous hybrid species and agamospecies described for the genera *Hieracium, Pilosella*, and *Taraxacum* [15]. These taxonomically diverse genera excluded, the tribe comprises approximately 1500 species. Based on a recent DNA-molecular phylogenetic study, the Cichorieae are divided into 11 subtribes [15]. Numerous species of the Cichorieae have been widely used for medicinal and food purposes since ancient times. *Taraxacum officinale* F.H.Wigg (Crepidinae), *Cichorium intybus* L. (Cichoriinae), and *Scorzonera hispanica* L. can serve as examples [4,5,14,16,17].

Plant taxonomic studies traditionally rely on morphology and karyology (e.g., [18,19]) as well as micro-morphological characters including those derived from pollen (e.g., [20,21]) and trichomes [22]. The taxonomy of the Asteraceae is mainly based on morphological characters like polysymmetric tubular florets characterising the Carduoideae and ligulate flowers characterising the Cichorioideae [3]. In contrast, taxonomic studies rarely deal with anatomical characters (e.g., [23]), i.e., the “internal morphology” of plants. Anatomy-based taxonomic studies in the Asteraceae mostly concentrated on aerial organs, seeds, and pollen [20,24,25,26,27]. As for the anatomy of the roots and rhizomes, the main differentiation of the two tribes is related to the internal secretory system: the presence of articulated laticifers in the phloem of the Cichorieae and the occurrence of schizogenous and lysigenous secretory ducts (i.e., resin ducts, which may be replaced by secretory cavities) in the Cardueae, structures missing with few exceptions in the Cichorieae [1].

Anatomical descriptions of underground organs are available for a couple of species from the Cardueae and Cichorieae (e.g., [7,28,29,30]), but most studies deal with species of South American provenance, in particular with species containing secretory ducts and cavities [31,32]. Anatomical studies on European species, especially from the tribe Cichorieae are, in contrast, rare [6,33] and comparative taxonomic studies are largely missing.

The availability of molecular phylogenetic hypotheses allows for reconstruction of the evolution of independent characters. In particular, the evolution of morphological characters has been reconstructed to get both a deeper understanding of the processes underlying the diversification of a given taxonomic group as well as the taxonomic value of characters [34,35,36]. These analyses thus allow reassessment of homology statements, traditionally used to define taxa, i.e., to identify reversals, convergence, or parallelisms in the evolution of characters [37]. Studies combining anatomical characters with molecular data, however, are largely missing. An exception is the study on orchids [38].

In the present work we assessed the taxonomic relevance and the evolutionary history of anatomical characters of subterranean organs in pharmaceutically used species and close relatives from the Cardueae and Cichorieae. Specifically, we addressed the following aims: (1) Do root and rhizome anatomy allow for discrimination of infratribal taxa (i.e., subtribes, genera, and species/subspecies), particularly with respect to suitability for identity and purity control in pharmacy? Which characters are most suitable for this purpose? (2) Does evolutionary diversification in root anatomy reflect the DNA-based phylogeny of the Cichorieae? We were specifically interested in the identification of anatomical features specific to monophyletic taxa.

## 2. Materials and Methods

### 2.1. Plant Material

We analysed 59 species classified into 34 genera, and 12 subtribes from the Cardueae and Cichorieae. One to six individuals were analysed per species, usually sampled from different geographic locations, 188 specimens in total. Material was collected during or shortly after antheses in the wild and from plants cultivated in the Botanical Garden of the Department of Pharmacognosy, University of Vienna (Table 1). Vouchers are deposited in the herbarium of the Department of Pharmacognosy, University of Vienna [WUP]. The plant material was taxonomically determined using floristic treatments covering the sampled geographic areas [39,40,41,42]. Nomenclature follows [2].

### 2.2. Anatomical Analysis

**Figure 1:**
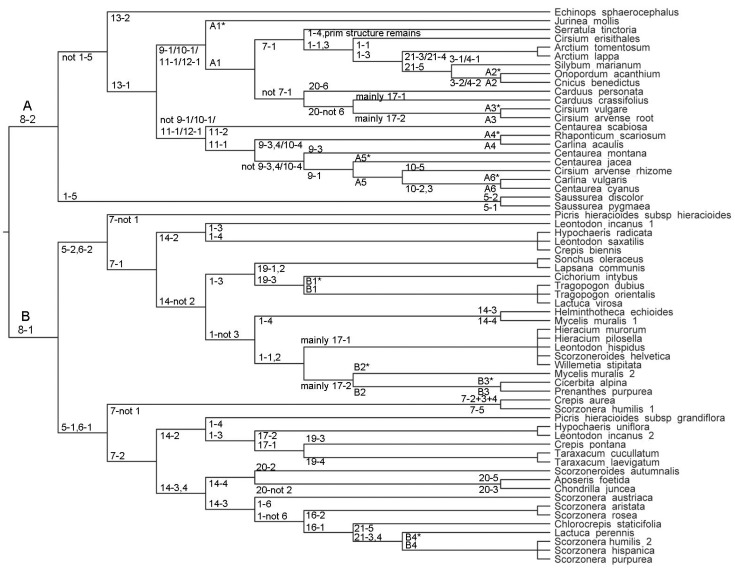
Binary decision tree based on anatomical characteristics of underground organs observed for 59 representatives of the Cardueae (branch A) and Cichorieae (branch B). Each informative node supports two branches joining taxa carrying alternative character states. Numbers refer to the character stated and defined in Table 3. Additional characters used are listed below. Species appearing twice in the tree carry the suffixes _1/_2. Short tip branches without assigned character states join species indistinguishable from each other based on the observed anatomical characters. A1*/A1 endodermal resin ducts multiple/not multiple the size of surrounding parenchyma cells; A2*/A2 diameter of largest vessels >100 µm/<100 µm; A3*/A3 cortex without/with aerenchyma; A4*/A4 SDs in fascicular/interfascicular position; A5*/A5 phytomelanin-coated sclereids present/absent; A6*/A6 secretory ducts with C1:C2 = 0.40−0.70/>0.9; B1*/B1 length: width of vessel elements <2/>3; B2*/B2 diameter of largest vessels <45 µm/>45 µm; B3*/B3 length: width of vessel elements <2/>3.5; B4*/B4 phellem special/regularly laminated.

Preparation of roots and rhizomes followed [7]. As standardization proved to be important [7], anatomical sections were taken (i) 1.5–2.5 cm below the insertion of the rosette leaves or below the basis of the superterranean shoot axis; (ii) from the longitudinal middle; and (iii) from the tip of the root. Sections were embedded in a few drops of chloral hydrate (60% in water) and observed using a Nikon Optiphot 2 light microscope and a Nikon Eclipse E 600 fluorescence microscope at 330–380 nm excitation. Images were taken using a Samsung Digimax V50 Digital Camera. Twenty-two nominal characters with multiple and binary character state expressions and one quantitative character were defined (Table 3). For computation of discriminant function analyses and construction of the binary decision tree (see below), multistate characters were converted into binary-coded variables (0 = absent; 1 = present), raising the total number of characters to 83 (including some additional anatomical characters, see Figure 1).

### 2.3. Discriminant Function Analyses and Binary Decision Tree

Discriminant function analyses (DFA) were performed on the basis of the whole dataset (188 accessions and 83 characters) as well as separately for the Cichorieae (115 accessions and 56 characters). Analyses were performed using Statistica^®^ and the general discriminant analytical model and step-wise forward [43]. Phi-coefficients were used to ensure that all highly correlated characters were excluded by the DFA algorithm correctly. For optimal performance of the DFA, sufficient variance in each selected taxon was requested. Therefore, subtribes (Hyoseridinae, Carduinae, Carlininae, Centaureinae, Cichoriinae, Chondrillinae, Crepidinae, Echinopsidinae, Hypochaeridinae, Hieraciinae, Lactucinae, and Scorzonerinae) instead of species were used as a categorical grouping variable. Specificity of anatomical characters on the level of subtribes was assessed manually. Taxon-specificity of anatomical characters on the level of genera and species was assessed in constructing a binary decision tree. Characters were chosen in terms of their unambiguousness and with the purpose of methodologically quick assessment (Figure 1).

### 2.4. DNA Extraction and Sequencing

Total genomic DNA was obtained from leaf tissue extracted from preserved herbarium specimens using the NucleoSpin^®^ 96 Plant II extraction kit (Macherey Nagel, Düren, Germany). For amplification of the nuclear ribosomal DNA (nrDNA), we used the forward primer 5′-GGA AGG AGA AGT CGT AAC AAG-3′ and reverse primer 5′-GGG TAA TCC CGC CTG ACC TGG-3′ [44]. The forward and reverse primer each carried a 5′-end extension complementary in sequence to the M13 forward (5′-GCA TGT TTT CCC AGT CAC GAC-3′) and the M13 reverse (5′-ACT TCA GGA AAC AGC TAT GAC-3′) primers, respectively, used in the cycle sequencing reaction. Sequences comprised part of the ITS1, the whole 5.8S rRNA gene, and part of the ITS2. Twenty-five µL PCR reactions were performed in a master mix containing 1× PCR buffer, 2.5 mM MgCl, 0.2 µM of each primer, 0.4 mM of each dNTP, 0.8 units DNA polymerase (Phire Hot Start II, Finnzymes, Espoo, Finland), and 10–100 ng of template DNA on a C1000 (Biorad, Hercules, CA, USA) thermal cycler. Thermal cycling started with a denaturation step at 98 °C lasting 1 min, followed by 35 cycles each of 10 s denaturation at 98 °C, 5 s annealing at 63 °C, and 15 s elongation at 72 °C. Amplification ended with a final hold at 10 °C. PCR products were purified using the NucleoFast Kit (Macherey-Nagel, Düren, Germany) and sequenced using the M13 primers. Cycle sequencing reactions were performed on both strands in a mix of 1.0 µL BigDye Terminator v3.1 (Applied Biosystems, Life Technologies, Waltham, MA, USA), 0.5 µL primer (10 µM), 2.0 µL 5× sequencing buffer, and 4.0 µL cleaned-up PCR product. The sequencing reaction started with a denaturation step at 96 °C lasting 1 min, followed by 35 cycles each of 10 s denaturation at 96 °C, 5 s annealing at 50 °C/48 °C for the forward/reverse primer, and 3 min elongation at 60 °C. Amplification ended with a final hold at 12 °C. Cycled products were cleaned by spinning through Sephadex G-50 Fine (5 g per 75 mL of ddH2O) (GE-Healthcare, Pittsburgh, PA, USA) and sequences were produced on a 3730 DNA Analyzer capillary sequencer (AB, Life Technologies) following the manufacturer’s protocol. PCR and sequencing were performed twice resulting in usually four but at least two sequences per specimen. Obvious sequencing errors were corrected based on the electropherograms and a consensus was made.

### 2.5. Alignment of DNA Sequences

Nuclear DNA sequences for taxa not sequenced here were obtained from NCBI (http://www.ncbi.nlm.nih.gov); accession numbers given in Table 1. In only two cases, sequences from closely related species were available and used as substitutes: *Leontodon saxatilis* Lam. was replaced by *Leontodon hispidus*, and *Saussurea pygmaea* by *Saussurea discolor*. Sequences were aligned using the program GeneDoc^®^ [45].

### 2.6. Phylogenetic Reconstructions

The phylogeny of the Cardueae and Cichorieae were separately reconstructed using *Helminthotheca echioides* and *Arctium lappa,* respectively, as outgroups. Some regions (listed in the “Results” part) within the completed alignment had to be excluded from further analysis because of ambiguous homology among sequences. Phylogenetic analyses were based on single nucleotide polymorphisms (SNPs). For the Cardueae, indels were 0/1 coded and added as additional characters at the end of the alignment. MrBayes 3.1 was used for Bayesian phylogenetic inference (http://mrbayes.csit.fsu.edu/index.php; [46]). The most likely DNA substitution models were selected for every tribe using ModelTest version 3.7 [47] and the Akaike Information Criterion. The alignments exclusive of the regions of ambiguous homology were saved in nexus format, the PAUP command block as included in the modelblockPAUPb10.txt-file of the Modeltest package was accordingly added and the completed files were executed in PAUP 4.0 version 4.0b10 [48]. The obtained output file (model.scores), containing a matrix of the log likelihood scores corresponding to the tested models, was finally executed in Modeltest under the default mode. The most likely model and the values of the estimated model parameters were entered into the input file of MrBayes as the specification of the evolutionary model. The following settings were used for the Cichorieae and Cardueae, respectively: prset revmatpr = dirichlet (0.70, 1.88, 1.74, 0.30, 4.08, 1.0), statefreqpr = dirichlet (0.25, 0.23, 0.23, 0.29), and revmatpr = dirichlet (0.68, 2.15, 1.64, 0.37, 6.49, 1.0), statefreqpr = dirichlet (0.22, 0.25, 0.29, 0.24). Coded gaps were treated as “Standard” datatype. The parameter nst was set to 6 and rates changed to invgamma. The analyses were run for 1,000,000 generations each with every 100th generation sampled and a temperature of 0.2. The obtained 10,000 samples of substitution model parameters, trees and branch lengths were summarized with a burnin of 2500 and graphically represented on the corresponding 50% majority rule consensus trees. The statistical support of tree clades was estimated using the posterior node probabilities calculated by MrBayes and in addition by a heuristic bootstrap analysis carried out under the parsimony optimality criterion (MP) using the same alignments/characters and PAUP 4.0b10 [47]. PAUP was run under its standard settings, except for the maximum number of retained trees and the number of bootstrap replicates which both were increased to 1000. Bootstrap values for clades which held identical sets of accessions in the maximum parsimony as well as in the Bayesian analysis were finally plotted onto the Bayesian 50% majority rule consensus tree.

### 2.7. Reconstruction of Character Evolution

The 50% majority rule consensus tree obtained from Bayesian analysis was taken to reconstruct the evolution of anatomical characters in the Cichorieae (not in the Cardueae which were phylogenetically poorly resolved) using Mesquite version 2.6 [49]. Ten multistate and six binary characters, which were variable within the tribe, (see Table 2: characters 8–13 are missing within the Cichorieae) were explored. Character states were treated as unordered. Evolution was reconstructed for root characters only (not for rhizome characters due to low sample size). A Mesquite input file in nexus format was created including a taxa block defining the taxon labels, a character block containing the coded variables, and a tree block providing the Bayesian 50% majority rule consensus tree. The file was executed in Mesquite under the parsimony method of ancestral character reconstruction using the TRACE CHARACTER HISTORY command. Conformity of the DNA-molecular and anatomical character evolution was assessed in calculating for each anatomical character the homoplasy index hi = 1 − ci. The consistency index ci_i_ = m_i_/s_i_ with m_i_ being the minimal number of changes required to transform the observed states of a character i into each other (calculated manually) and s_i_ denoting the actual number of parsimonious character changes along the phylogenetic tree (calculated using Mesquite). The overall consistency index (hi = 1 − Σm_i_/Σs_i_) calculated over all i was compared to the hi of the DNA-molecular phylogeny obtained performing a MP analysis using PAUP (settings as mentioned before).

## 3. Results

### 3.1. Sequence Variation

The length of the alignment including all taxa and comprising the 5.8S RNA gene and parts of ITS1 and ITS2 was 682 bp. We encountered intragenomic variation (polymorphic sites seen as superimposed peaks in the electropherograms) in sequences from three species: one site in *Carlina personata* and *Taraxacum cucullatum* and seven sites in *Picris hieracioides* subsp. *hieracioides*. These ambiguous base-callings were coded using IUPAC (International Union of Pure and Applied Chemistry) ambiguity codes. After exclusion of regions of ambiguous alignment (alignment positions 471–496 and 91–100, 457–496 for the Cardueae and Cichorieae, respectively) the effective lengths of the alignments were reduced to 629 bp (Cardueae) and 631 bp (Cichorieae). The following measures were deduced from these reduced alignments for the ingroups and—in parentheses—the entire datasets including the outgroup. Cardueae: 256 (287) sites were variable. Of these, 160 (169) sites were parsimony informative. In addition, 11 (12) out of 33 indels included in the phylogenetic analyses were parsimony informative. Cichorieae: 320 (328) sites were variable and 266 (272) sites were parsimony informative. Twenty-four (27) out of the 61 (63) indels were parsimony informative but not included in the phylogenetic reconstruction. Sequences were assembled within this study and submitted to GenBank (accession numbers KM262846–KM262853). Genbank accession numbers of sequences, including published ones, are given together with the taxa names in Table 1.

### 3.2. Phylogenetic Relationships

**Figure 2:**
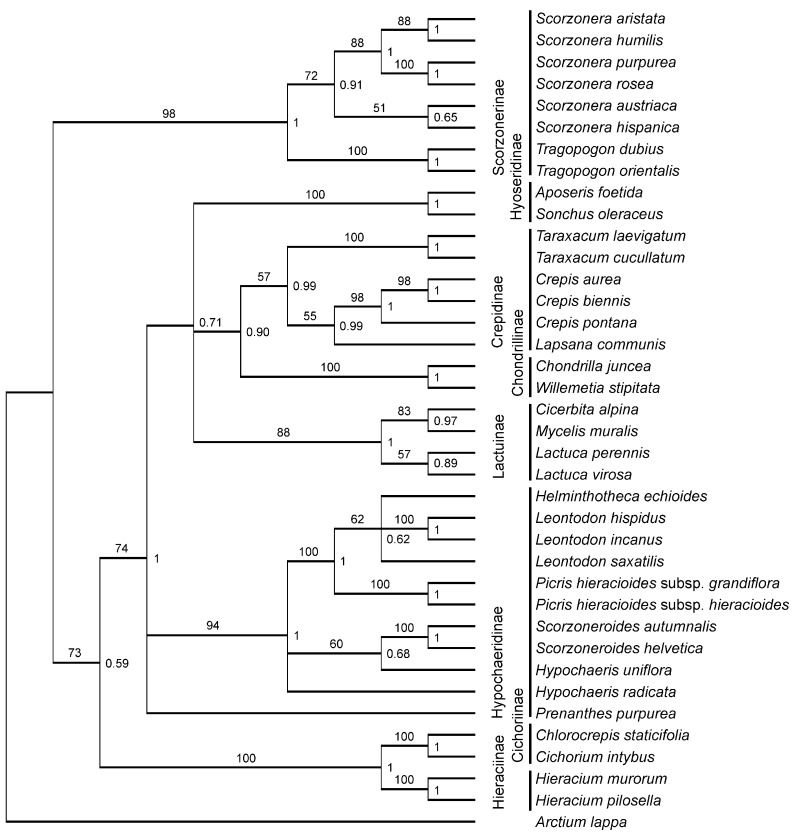
50% majority rule consensus tree of 37 species representing eight subtribes and 22 genera from the Cichorieae based on nrDNA single nucleotide polymorphisms. The tree was reconstructed from 7500 retained trees (out of 10,000; burnin = 2500) inferred using MrBayes. Posterior probabilities are given to the right of the nodes. Numbers above branches are bootstrap values obtained for taxonomically equivalent clades running an independent maximum parsimony analysis on the same character set and accessions using PAUP.

The various subtribes of the Cichorieae were resolved in the consensus tree of the Bayesian phylogenetic reconstructions (Figure 2) as monophyletic lineages, save the Hypochaeridinae. The Hypochaeridinae formed two separate phylogenetic lineages (*Prenanthes pupurea* L. and the remaining members of the subtribe) which were part of a polytomy additionally supporting a clade joining the Lactucinae, Hyoseridinae, Crepidinae, and Chondrillinae. The monophyly of subtribes was supported by bootstrap values (obtained from the heuristic MP analyses) and Bayesian posterior probabilities ranging from 57–100 and 0.99–1.0, respectively. Genera (studied for two or more species) were monophyletic except for *Hypochaeris* (paraphyletic) and *Leontodon* (forming two lineages in a polytomous clade). All tree nodes received some statistical support with the weakest support being 0.52 posterior probability (found for a basal node within the Hypochaeridinae).

Compared to the Cichorieae, the phylogenetic relationships among the Cardueae were less resolved in the Bayesian consensus tree (Figure S1): a basal polytomy joined the Carlininae, Echinopsinae (studied for *Echinops sphaerocephalus* only), two lineages of the Carduinae and another polytomous clade supporting (as separate lineages) the Centaureinae and three other members of the Carduineae (*Arctium*, *Jurinea mollis*, and *Saussurea discolor*). Polytomies further existed at the basis of the Centaureinae and within the clade joining the majority of the Carduinae taxa. Subtribes and genera (studied for two or more representatives) were monophyletic (with bootstrap values and posterior probability supports of 100 and 1, respectively), save the Carduinae, Centaurea, and Cirsium, which were paraphyletic.

### 3.3. Evolution of Anatomical Characters

**Figure 3:**
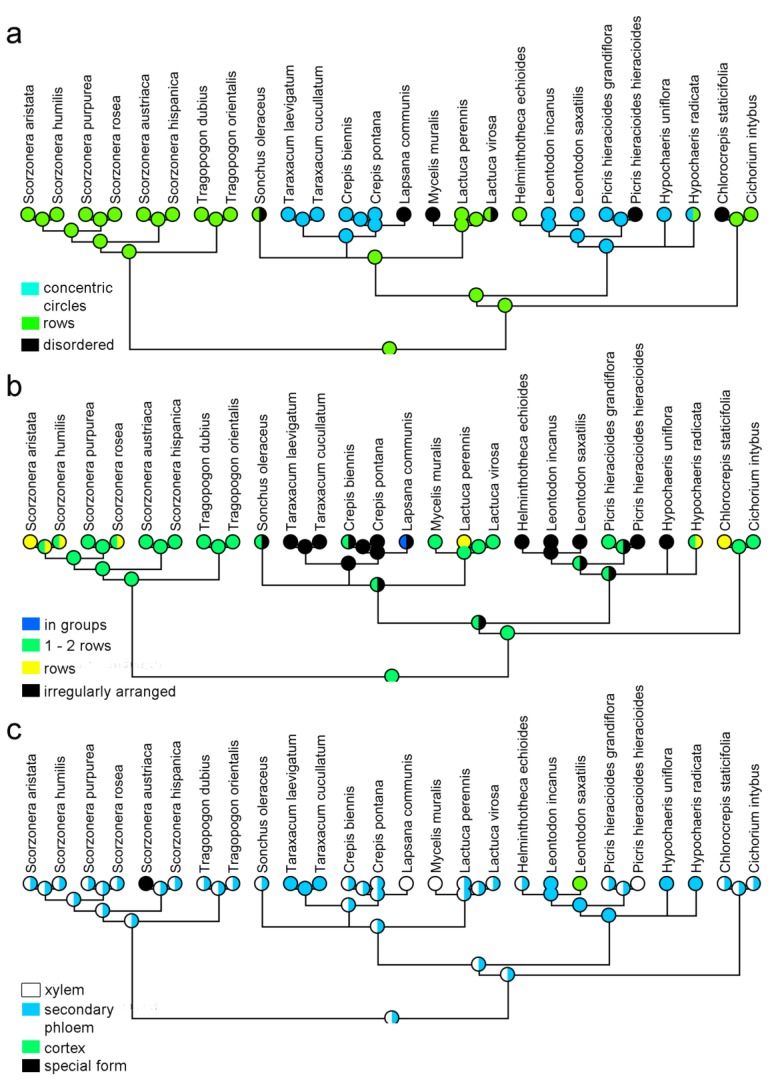
Three examples of ancestral state reconstructions based on the 50% majority rule consensus from Figure 2. Character states of ancestral nodes were inferred using Mesquite under the parsimony criterion: (**a**) arrangement of laticifers; (**b**) vessel arrangement; and (**c**) tissue dominating in root cross-section. Character states are indicated in the legend. See the text for a discussion of the evolution of the characters and their suitability for the characterization of phylogenetic lineages.

The evolution of anatomical root characters was traced for the phylogenetically well-resolved Cichorieae only (Figure 3a–c, Figure S2). The parsimonious reconstructions revealed high degrees of homoplasy (hi = 0.33−0.88 for the individual characters: Table 2). Lowest values were obtained for the arrangement of vessels (hi = 0.57) and the occurrence of phellem (hi = 0.33). The overall hi was 0.66 compared to 0.59 obtained in the DNA-molecular MP analyses.

### 3.4. Anatomical Differentiation of Taxa

Fifty-six out of the 59 analysed species were assigned to single branches in the binary decision tree; three species appeared twice in the tree. *Cirsium arvense* was entered for both root as well as rhizome anatomy. Five dichotomies, three tritomies, and one pentatomy (positioned at branch tips) joined taxa indistinguishable from each other based on the analysed set of anatomical characteristics. Accordingly, the tree comprised 46 binary decisions (Figure 1) and distinguished 34 species/subspecies by a unique combination of hierarchically arranged characters. The most basal split separated the Cardueae from the Cichorieae. Except for *Arctium*, *Hieracium*, *Saussurea*, *Taraxacum,* and *Tragopogon,* genera analysed for at least two species as well as invariably all subtribes were assigned to different branches. Indistinguishable taxa do not necessarily belong to the same genus, e.g., *Sonchus oleraceus* and *Lapsana communis*; *Crepis biennis*, *Hypochaeris radicata*, and *Leontodon saxatilis*. *Taraxacum* and *Arctium* represent examples for missing infrageneric variability. In contrast, the genus *Scorzonera* showed variability in phellem structure.

Thirty-six out of the 83 binary characters were invariable in all individuals of at least one subtribe. Such constantly expressed characters were observed for all subtribes, save the Centaureinae with the number of characters ranging from four (Carduinae) to 21 (Echinopsidinae). However, only one of these characters (secondary cavities) was subtribe-specific (Echinopsidinae). Furthermore, save the Echinopsidinae, none of the subtribes could be characterised by a unique combination of constantly expressed characters, although 19 not-constantly expressed characters (i.e., expressed in only some representatives) were specific to six out of 12 subtribes.

**Figure 4:**
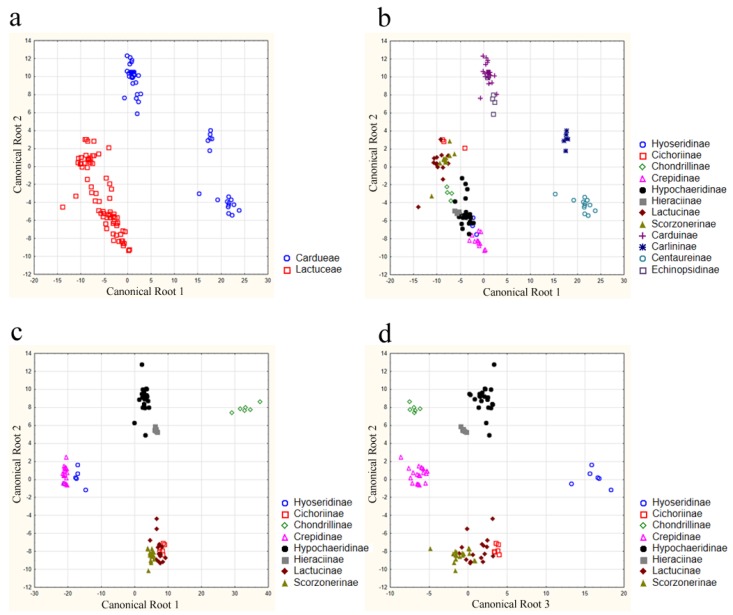
Results of DFA computations of (**a**) the Cardueae and the Cichorieae; (**b**) the Cardueae and the Cichorieae displaying their subtribes; (**c**) the Cichorieae showing canonical root 1, 2; (**d**) the Cichorieae showing the canonical roots 2, 3; only based on binary anatomical root and rhizome characters. Legends differentiate sampled subtribes.

For the computation of discriminant function analyses (DFA), all characters were dichotomized. This led to 83 binary characters. In a first step, a computation of all phi-coefficients was performed. Several computations were performed to answer our introductory questions. The phi-coefficients were used to ensure that all highly correlated characters were excluded by the DFA algorithm correctly. Table 4 summarizes the results of the various analyses (see also Figure 4). The DFA number 2, based on all examined species, separated the subtribes with a classification probability of 99.5%, reasoned on the “misplacement” of one sample from *Prenanthes purpurea* to the Lactucinae, primarily assigned to the Hypochaeridinae. However, all species of the Cichorieae were classified according to their subtribal affiliation in a separate DFA (number 4 and 5 in Table 4). The DFA from the subtribes of Cardueae shows one misclassification (*Onopordum acanthium*).

## 4. Discussion

### 4.1. Taxonomic Value of Anatomical Root and Rhizome Characters

We uncovered in our study a striking difference in the discriminative value of anatomical root and rhizome characters with a hierarchical level of taxa. The anatomy proved to be valuable to discriminate tribes and numerous species but was only of restricted value to distinguish subtribes and genera from each other.

The Cardueae and Cichorieae were discriminated by the occurrence of resin-secreting structures and lacticifers, respectively, as already recognised by [8,25,25,51,52,53]. In contrast, the discrimination of subtribes was less obvious. The DFA number 2—based on the whole data set—separated all subtribes although showing a “misplacement” of *Prenanthes purpurea*. One sample of this species was assigned to the subtribe Lactucinae. The problematic circumscription of the genus *Prenanthes* is discussed in detail in Wang et al. [54]. The authors propose the assignment of *Prenanthus purpura* to the subtribe Latucinae. Consequently, for the DFA computation number 3, the assignment of *Prenanthes purpura* was changed from Hypochaeridinae to Lactucinae resulting in the resolution of the misplacement.

Nevertheless, solely in one case, a single anatomical character (secretory cavities of the Echinopsidinae) unequivocally characterised a subtribe. Moreover, it remains to be proven whether the discriminate value of this character would hold within a larger data set. Save the Centaureinae, the anatomical characters constantly expressed within subtribes were observed for all of these, but were not subtribe-specific. Consequently, they don’t allow on their own for unambiguous discrimination of these taxa. Examples are multiseriate medullary rays (more than five cells in width) and laticifers arranged in radiant rows which were both constantly expressed in the Scorzonerinae, but occurred in other subtribes too (Table S3).

As for identity and purity control in pharmacy, discrimination on the species level is the most relevant. We identified three different situations with respect to the value of anatomy for species discrimination: (i) species distinguishable based on a single character or a unique combination of character states; (ii) variable but still species-specific combinations of character states; and (iii) indistinguishable species.

Specific characteristics allowing for quick identification of species were for instance interxylary cork in *Saussurea discolor* and *S. pygmaea* (see also [33]) and irregular secondary growth in *Scorzonera austriaca* (see [9]). In the majority of cases, however, a combination of characters was needed for species identification. As illustrated in the binary decision tree (Figure 1), either few features defined a species (e.g., *Crepis aurea*: fibers missing + sclereids present in cortex, secondary phloem and xylem) or considerably high numbers of characters had to be established as for instance in *Cicerbita alpina* and *Prenanthes purpurea*. This pair of species could be distinguished in the last instance based on the length: width ratio of vessel elements (*Cicerbita alpina* <2; *Prenanthes purpurea* >3.5), but several additional characters had to be assessed too. Importantly, identification was not restricted to species from different genera, but species discrimination was also possible within genera (e.g., Centaurea or Cirsium).

In some cases, character expression varied within a single species resulting in more than one character combination needed to be considered for identification, as demonstrated by *Leontodon incanus*, *Mycelis muralis,* and *Scorzonera humilis*. Nevertheless, unambiguous species discrimination remained possible.

Although the majority of species could be distinguished from each other, discrimination of species from the same genus couldn’t be accomplished in all cases: e.g., *Taraxacum* spp., *Arctium* spp., *Tragopogon* spp. Likewise, some species from different genera were indistinguishable, for instance *Hypochaeris radicata*, *Leontodon saxatilis*, and *Crepis biennis*; *Sonchus oleraceus* and *Lapsana communis;* or *Scorzonera aristata* and *Podospermum rosea* (the latter formerly belonging to the genus Scorzonera).

Table S3 summarizes anatomical character states observed for the studied species (see also [7,9]) and is intended for use in identity control. All things considered, the identification of pharmaceutically important species (*Arctium* spp., *Carlina acaulis*, *Cichorium intybus*, *Cnicus benedictus*, *Silybum marianum*, *Taraxacum* spp.) from root cross-sections was possible. However, since physical integrity of the sample is mandatory to maintain the anatomical context, identification from powdered drugs appears rather difficult or impossible for the majority of species.

### 4.2. DNA-Molecular Phylogenies and Anatomical Character Evolution

Considering statistically supported clades only (bootstrap values ≥ 50, posterior probabilities ≥ 0.5), phylogenetic relationships among genera inferred for the Cichorieae were largely congruent with the nrDNA-based reconstructions by Kilian et al. [16]. Topological conflicts existed with the placement of *Helminthotheca* as a sister to *Picris* in Kilian et al.’s study and as a sister to *Leontodon* in our reconstruction. In addition, *Scorzoneroides* was basal to the clade carrying *Hypochaeris* in the earlier study, whereas it grouped with *H. uniflora* in the present one. However, placement of both genera was rather weakly supported in Kilian et al.’s phylogeny (posterior probabilities of 0.5 and no bootstrap support).

The majority of subtribes and genera were monophyletic, indicating that the DNA molecular-based phylogeny of the Cardueae and Cichorieae largely corroborates their current taxonomy [10,15]. The reconstruction of the evolution of anatomical root characters constrained on the molecular phylogeny of the Cichorieae revealed homoplasy for most characters (Table 2), meaning that parallel origins and/or reversal of states occurred for almost all characters. As a consequence, synapomorphies (i.e., a derived character defining a monophyletic group) qualifying as diagnostic anatomical root characters for both genera and subtribes were missing.

An example for a derived anatomical character state is the concentric arrangement of laticifers, which is, according to our analysis, an evolutionary modification of the ancestral radial arrangement (Figure 3a). The concentric arrangement is a synapomorphy of both the Crepidinae and the Hypochaeridinae. Although an anatomically conspicuous feature, parallel origins disqualified the concentric arrangement of laticifers as an independent diagnostic character state. Another analogous illustrative example is the parallel origin of multilayered phellem in *Scorzonera* and *Lactuca* (Figure S2). However, the optional origin of irregular vessel arrangement (Figure 3b) and of faint pits (Figure S2) at the basis of the clade joining the Hyoseridinae, Crepidinae, Lactucinae, and Hypochaeridinae may constitute autapomorphies (i.e., derived unique characters defining a monophyletic group or tip branch), thereby supporting the DNA-molecular-based phylogeny. The potential value of anatomical characters in phylogenetic reconstruction is also seen from homoplasy indices lower than the overall hi inferred for the molecular reconstruction for some anatomical characters (Table 2). Similar patterns have been demonstrated (although with different degrees of homoplasy) for other anatomical [38,55] as well as morphological characters (e.g., [56]). Coincidence of the molecular and anatomical phylogenetic signal has also been demonstrated by [57], who found a basal lineage joining the majority of the Maloideae taxa (Rosaceae) supported by both nrDNA and a type of sclereids arrangement in fruit flesh. Another example is the study of root anatomy of the subfamily Cranichideae (Orchidaceae) by [50], who found tilosome distribution phylogenetically informative.

However, our results demonstrated a difference in the evolutionary dynamics of state changes among characters and importantly in phylogenetic information of characters. A conspicuous feature in the evolution of anatomical root characters was the high frequency of parallel origins and reversals of character states observed within all subtribes and, to a lesser extent, genera. Variability in the character state was observed both among as well as within species and can be explained by replacement of character states as anatomical differences in time (i.e., as a result of the evolutionary fixation of character changes) and space (i.e., variation among extant individuals), respectively. Examples for complex patterns of character distribution due to intraspecific variation are the dominance of tissues in root cross-sections (Figure 3c) and vessel perforations (Figure S2). The extent of tissue is a quantitative character, and differential promotion vs. suppression of tissues may explain the high intra- and interspecific variability. In spite of that variation among and within species dominating the picture, uniform character expression was observed for the Scorzonerinae (eight uniform characters), Crepidinae (7), Lactucinae (4), Hypochaeridinae (3) as well as the Cichoriinae (11). Since only a limited number of the known species and genera of the Cichorieae could be included in this study, the inferred degrees of character homoplasy will be underestimated.

## Figures and Tables

**Table 1:** Subtribal affiliation and collection history of 188 studied specimens from the Cardueae and Cichorieae. Specimen identifiers are provided in brackets following the geographic origin. GenBank accession numbers of DNA sequences are provided in the last column. Taxonomy follows [50]. Plant material collected by Christoph Dobeš (CD), Elisabeth Ginko (EG), Günther Stadler (GS), Johannes Saukel (JS), Silvia Fialová (SF), Valerie Klatte-Asselmeyer (VK), and Werner Lahner (WL). Vouchers are deposited in herbarium WUP.

Subtribe	Species	Origin and Collection History	GenBank Accession No.
Echinopsinae	*Echinops sphaerocephalus* L.	Austria, Vienna, EG (ES02-09, ES03-09)	AY538637
		Austria, Lower Austria, Tullnerbach, EG (ES06-09)	
		Austria, Lower Austria, Karnabrunn, CD (ES07-09)	
		Austria, Tyrol, Fließ, VK (ES08-09)	
Carlininae	*Carlina acaulis* L*.*	Austria, Vienna, EG (CA02, CA05); Germany, Baden-Württemberg, Schwäbische Alb, Herbarium of Hohenack, Nr. 652 (CA01, CA04); commercially sample, Kottas Pharma, 1881, 1882; DNA-molecular analysis: Austria, East Tyrol, Lienz, CD (AST26)	KM262847
	*C. vulgaris* L.	Poland, Gutkowo, Olsztyn, EG (CV02-09–CV05-09)	AY826246
Carduinae	*Arctium lappa* L.	Austria, Vienna, EG (AL01-09, AL05-09, AL3A-09)	FJ528300
	*A. tomentosum* Mill.	Austria, Traiskirchen, EG (AT01-10–AT03-10)	GQ281034
	*Carduus defloratus* L.	Austria, Lower Austria, Gippel, CD (CC01-08)	AY826241
		Austria, Lower Austria,	
	*C. personata* (L.) Jacq.	Austria, Styria, Schneealpe, EG (CaP01-09–CaP03-09;	KM262846
		DNA-molecular analysis: CaP03-09)	
	*Cirsium arvense* (L.) Scop.	Austria, Vienna, EG (CiA02-08); Slovakia, Modra, SF (CiA01-08)	AF443680
		USA, New York, Peekskill, Planta America Septentrionalis, Le Roy (CiA03-08)	
	*C. erisithales* (Jacq.) Scop.	Austria, Styria, Schneealpe, EG (CE01-09–CE03-09;	KM262848
		DNA-molecular analysis: CE01-09)	
	*C. vulgare* (Savi) Ten.	Poland, Gutkowo, Olsztyn, EG (CiV01-09–CiV03-09)	AF443715
	*Jurinea mollis* (L.) Rchb.	Austria, Vienna, EG (JM01-09)	AY780404
		Austria, Burgenland, Winden, JS (JM03-86, JM04)	
	*Onopordum acanthium* L.	Austria, Lower Austria, Buchberg, WL (O01-08, O02-08);	AY914827
		Italy, Southern Tyrol, Vinschgau, CD (AST3)	
	*Saussurea discolor* (Willd.) DC.	Austria, Carinthia, Lesachtal, JS (SD01-09–SD07-09)	AF319146, AF319092
	*S. pygmaea* Dunn	Austria, Styria, Schneealpe, EG (SaP01-09–SaP06-09, SaP S1)	substituted with* S. discolor* (see *Materials and Methods*)
	*Silybum marianum* (L.) Gaertn.	Austria, Lower AUT, Buchberg, WL (MD1-08, MD2-08, SM1); Slovakia, Botanical Garden of Bratislava, SF (MD3-08)	AY914831
Centaureinae	*Centaurea jacea* L.	Austria, Karnabrunn, CD (CJ01-09, CJ02-09)	AM114332
		Austria, Vienna, EG (CJ8-1)	
	*Cyanus segetum* Hill. Synonym:* C. cyanus* L.	Austria, Vienna, EG (CS3-08)	AY826254
		Germany, Baden-Württemberg, Kronau, CD (CS2-08)	
		Poland, Mazury, Zabie, EG (CS1-08)	
	*Cyanus montanus* (L.) Hill. Synonym:* C. montana* L.	Austria, Lower Austria, Unterberg, EG (C01-08)	L35887
		Austria, Lower Austria, Pressbaum, EG (C03-08); Slovakia, Modra, SF (C02-08)	
	*C. scabiosa* L.	Austria, Vienna, JS (CS03-08); Poland, Gutkowo, Olsztyn, EG (CS02-08); Switzerland, Graubünden, Lavin, CD (CS01-08A, CS01-08B)	FJ459692
	*Centaurea benedicta* (L.) L. Synonym: *Cnicus benedictus* L.	Botanical Garden of the Department of Pharmacognosy, University of Vienna (CB1—S3,S4)	DQ319091
	*Rhaponticum scariosum* Lam.	Liechtenstein, Saminatal, GS (RS01-09, RS02-09)	DQ310951
	*Serratula tinctoria* L.	Austria, Vienna, JS (ST01-09–ST04-09)	AJ868085, AJ868086
Scorzonerinae	*Scorzonera aristata* Ramond ex DC.	Austria, Carinthia, Lumkofel, JS (SAr01-09–SAr03-09)	AY508192
	*S. austriaca* Willd.	Austria, Vienna, EG (SA01-09, SA04-09, SA05-09)	AM117047
	*S. hispanica* L.	Austria, Lower Austria, Anninger, JS (SHI01)	AJ633472
		Austria, Lower Austria, Weinling, JS (SHI02, SHI03)	
	*S. humilis* L.	Austria, Lower Austria, Drosendorf, JS (SH02, SH03)	AJ633476
		Austria, Lower Austria, Bisamberg (SH01)	
	*Podospermum purpureum* (L.) W.D.J.Koch & Ziz Synonym: *S. purpurea* L.	Austria, Vienna, EG (SP01-09–SP03-09)	AM117054
	*Podospermum* *roseum*(Waldst. & Kit.) Gemeinholzer & Greuter Synonym:* S. rosea* Waldst. & Kit.	Austria, Carinthia, Lumkofel, JS (SR01-09, SR02-09; DNA-molecular analysis: SR02-09)	KM262852
	*Tragopogon dubius* Scop.	Austria, Vienna, EG (TD1-08);	AY645813
		Italy, Vinschgau, Schluderns, CD (AST1)	
	*T. orientalis* L.	Austria, Lower Austria, Araburg, EG (TO01-08)	AY508170
		Austria, Lower Austria, Laaben, EG (TO02-08)	
		Poland, Gutkowo, Olsztyn, EG (TO03-08)	
Lactucinae	*Lactuca* *alpina*(L.) A. Gray Synonym: *Cicerbita alpina* Wallr.	Austria, Styria, Schneealpe, EG (CiAl01-09–CiAl03-09)	AJ633324
	*Lactuca perennis* L.	Italy, Eisacktal, JF (LP01)	AJ633334
		Italy, Southern Tyrol, Vinschgau, CD (LPAST14A, LPAST14B)	
	*L. virosa* Habl.	Austria, Vienna, EG (LV01-07, LV02-07)	AJ633335
	*Lactuca muralis* (L.) Gaertn. Synonym: *Mycelis muralis* (L.) Dumort.	Austria, Vienna, EG (M01-08, MM01-08)	AJ633338
		Austria, Lower Austria, Irenental, EG (M02-07)	
		Austria, Rekawinkel, EG (M01-07)	
Hyoseridinae	*Aposeris foetida* (L.) Cass. ex Less.	Austria, Carinthia, Feistritz im Rosental, JS (AF1, AST19)	DQ451822
		Germany, Garmisch-Partenkirchen, Wank, CD (AST23)	
	*Sonchus oleraceus* (L.) L.	Austria, Vienna, EG (SO01-07)	AY862581
		Austria, Lower Austria, Irenental, EG (SO02-07A, SO02-07B); Italy, Southern Tyrol (CD): seeds planted in Botanical Garden of the Department of Pharmacognosy (AST18)	
Crepidinae	*Crepis aurea* (L.) Cass.	Austria, Salzburg, Mehrlhütte, JS (CrA01-08–CrA04-08)	AF528483
	*C. biennis* Lapeyr.	Italy, Southern Tyrol, Vinschgau, CD (AST5)	DQ451818
		Slovakia, Modra, SF (CB01-08)	
		Botanical Garden Karl Franzens Universität Graz	
		Mürzsteger Alpen; Seewirtgraben/Mariazell (70)	
		Botanical Garten Berlin-Dahlem; DE-0-B-0164479: Hessen, Werra-Meißner-Kreis, Eschwege, leg. Royl & al. (814)	
	*C. pontana* (L.) Dalla Torre	Austria, Carinthia, Lesachtal, Lumkofel, JS (CP01-09–CP04-09; DNA-molecular analysis: CP07-09)	KM262849
	*Lapsana communis* L.	Italy, Southern Tyrol, Vinschgau, CD (AST17)	AJ633285
		Austria, Vienna, EG (LC01-08, LC02-08B); Germany, Baden-Württemberg, Blankenloch, CD (LC02-08A)	
	*Taraxacum cucullatum* Dahlst.	Austria, Salzburg, Riedingtal, JS (TC01-08–TC04-08; DNA-molecular analysis: TC04-09)	KM262853
	*T.erythrospermum* Andrz. ex Besser. Synonym: *T. laevigatum* DC.	Austria, Vienna, JS (TL01-08–TL03-08)	AJ633288
Chondrillinae	*Chondrilla juncea* L.	Austria, Vienna, Kovats (CJ02); Hungary, Holuby (CJ01)	AJ633348
	*Willemetia stipitata* (Jacq.) Dalla Torre	Austria, Salzburg, Maria Pfarr, JS (WS01-08–WS04-08)	EU436697
Hypochaeridinae	*Helminthotheca echioides* (L.) Holub	Botanical Garden of the Univ. Bonn; 1744	AF422123
		DE-0-Bonn-23773: F. Klingenstein & J. Manner,1998, Deutschland, Nordrhein-Westfalen, Bonn-Ippendorf (HE1)	
	*Hypochaeris radicata* L.	Poland, Gutkowo, Olsztyn, EG (5-100, 5-16)	EF107656
		Botanical Garden Berlin-Dahlem; 916	
		DE-0-B-2421281: Bayern, Kreis Wunsiedel, Fichtelgebirge, leg. Hempel; Italy, Eisacktal, Blumau-Rielinger-Atzwang, JS (H113)	
	*H. uniflora* Vill.	Austria, Salzburg, Lungau, JS (V310, V312)	AF528481
		Austria, Salzburg, Kareck, JS (101, HU01-08)	
	*Leontodon incanus* (L.) Schrank	Austria, Burgenland, St. Margarethen, JS (L100)	DQ451772
		Austria, Styria, Oberwölz, JS (L108)	
		Austria, Lower Austria, Baden, JS (L305)	
		Austria, Lower Austria, Anninger, JS (L358, L359)	
	*L. hispidus* L.	Austria, Lower Austria, Rekawinkel, EG (L1-106)	AF528485
		Austria, Lower Austria, Rax, EG (3-22)	
		Austria, Carinthia, Pöllatal, JS (L105)	
	*L. saxatilis* Lam.	Botanischer Garten Marburg; Akznr. 2000/77	substituted with *L. hispidus* (see *Materials and Methods*)
	*Picris hieracioides* L. subsp. *hieracioides*	Austria, Vienna, EG (PH02-07A, PH02-07B); Poland, Gutkowo, Olsztyn, EG (PH01-07); DNA-molecular analysis: PH03-07)	KM262851
	*P. hieracioides* subsp. *grandiflora* (Ten.) Arcang.	Botanical Garden of the Friedrich-Schiller-Universität Jena;	KM262853
		Weimar: Oberweimar (PHS4)	
	*Prenanthes purpurea* L.	Austria, Styria, Schneealpe, EG (PP01-09–PP03-09)	AJ633342
	*Scorzoneroides autumnalis* (L.) Moench	Poland, Gutkowo, Olsztyn, EG (L102–L104)	AF528486
	*S. helvetica* (Merat) Holub	Austria, Styria, Etrachtal, JS (L109)	DQ451766
		Austria, Styria, Prebertal, JS (L112)	
		Austria, Styria, Anger, Lessachwinkel, JS (L109)	
Hieraciinae	*Hieracium murorum* C.B.Clarke	Austria, Lower Austria, Hohe Wand, EG (HM01-08)	AF528492
		Switzerland, Graubünden, Bos-cha, CD (AST11)	
		Botanischer Garten Karl Franzens Universität Graz;	
		81; Schillingsdorf, VI-X/2007 (AD) (HM1)	
	*Pilosella* *officinarum* Vaill. Synonym: *H. pilosella* L.	Italy, Southern Tyrol, Vinschgau, CD (AST6); Poland, Gutkowo, Olsztyn, EG (01-07); (HP01-86)	AY879161
Cichoriinae	*Tolpis staticifolia*(All.) Sch.Bip. Synonym: *Chlorocrepis staticifolia* (All.) Griseb.	Italy, Southern Tyrol, Vinschgau, CD (AST7)	AJ633437
		Italy, Valbruna, CD (AST22)	
	*Cichorium intybus* L.	Austria, Lower Austria, Pressbaum, EG (CI1-07);	AY504694
		Austria, Vienna, EG (CI1-08); Poland, Gutkowo, Olsztyn, EG (CI2-07); Seeds of the Botanical Garden Berlin-Dahlem: DE-0-B-2003105: Brandenburg, Falkensee, leg. Dürbye 3090 (CI1)	

**Table 2:** Homoplasy indices (hi) for 15 anatomical root characters inferred from their evolution along the nrDNA 50% majority rule consensus tree of the Cichorieae in Figure 2. Numbers in brackets refer to the characters listed in Table 3.

Anatomical Character	*hi*
Tissue dominating in extension (2)	0.81
Cortex durability (3)	0.86
Endodermis (4)	0.88
Fibers in xylem (5)	0.86
Fibers in secondary phloem (6)	0.00
Sclereids (7)	0.67
Arrangement of laticifers (14)	0.60
Phellem (15)	0.33
Phellem cells (16)	0.67
Vessel perforation (17)	0.88
Vessel perforation (limited to: just reticulate—others)	0.80
Pits of vessels (18)	0.76
Medullary rays (19)	0.62
Arrangement of vessels (21)	0.62
Arrangement of vessels (limited to: linear arranged/in rows/irregular)	0.57
Minimum value	0.00
Maximum value	0.88
Mean value	0.66

**Table 3:** Quantitative and qualitative anatomical characters and their states defined to characterise the anatomy of subterranean organs in 59 representatives of the tribes Cardueae and Cichorieae.

Quantitative Character
Maximum Diameter of Vessels (µm)
**Qualitative Characters**
1. Type of subterranean organ—1-rhizome 2-rhizome part of taproot 3-taproot 4-fibrous root system 5-interxylary cork results in splitting of the root into various strands [34] 6-irregularly secondary growth: bundles of phloem and xylem separated by a cambium are irregularly dispersed over the transverse section [9]
2. Tissue dominating in extension—1-xylem 2-secondary phloem 3-cortex 4-special form (see character 1–6, [9])
3. Cortex durability—1-not enduring 2-enduring
4. Endodermis—1-not visible 2-clearly visible
5. Fibers in secondary xylem—1-missing 2-present
6. Fibers in secondary phloem—1-missing 2-present
7. Sclereids—1-missing 2-within cortex 3-within secondary phloem 4-within xylem 5-within phellem 6-within pith
8. Endodermal resin ducts—1-missing 2-present
9. Secretory ducts of type SD1 (C1: C2 of <0.3 [8])—1-missing 2-within secondary phloem fascicular 3-within secondary phloem interfascicular 4-within secondary xylem interfascicular
10. Secretory ducts of type SD2 (C1:C2 of >0.4 [8])—1-missing 2-within secondary phloem fascicular 3-within secondary phloem interfascicular 4-within secondary xylem interfascicular 5-within pith
11. Secretory ducts of type SD3 (lysigenous development [ 8])—1-missing 2-within secondary phloem fascicular
12. Secretory ducts of type SD4 [8]—1-missing 2-within cortex, secondary phloem and xylem 3-within cortex, secondary phloem and pith
13. Secretory cavities—1-missing 2-present
14. Arrangement of lacticifers—1-missing 2-in concentric circles 3-radiant rows 4-disordered
15. Phellem—1- 1 to 5 layers 2-multilayered 3-layers not enduring 4-missing
16. Phellem cells—1-thin-walled 2-thick-walled 3-missing
17. Vessel perforation—1-reticulate 2-pitted
18. Pits of vessels—1-simple pits 2-pits with faint border 3-pits with conspicuous border 4-missing
19. Medullary rays—1-uni- or biseriate 2-up to 5 rows 3-more than 5 rows 4-not visible
20. Pith cells—1-missing 2-cell walls thin-walled 3-cell walls slightly thickened 4-irregularly thickened 5-nodularly thickened 6-strongly thickened and pitted
21. Arrangement of vessels—1-in circles 2-in groups below or next to each other 3-in rows (1–2) 4-in rows (>2) 5-irregular
22. Crystal needles—1-missing 2-present

**Table 4:** Overview of DFA computations (number 1–7).

1	Discrimination possibility between the tribes Cardueae and Cichorieae (2 groups, 83 characters)→100% discrimination
2	Discrimination possibility between all subtribes of investigated Cardueae and Cichorieae (12 groups, 83 characters). *Prenanthes purpurea* as a member of the subtribe Hypochaeridinae→99.5% discrimination
3	Discrimination possibility between all subtribes of investigated Cardueae and Cichorieae (12 groups, 83 characters). *Prenanthes purpurea* as a member of the subtribe Lactucinae→100% discrimination
4	Discrimination possibility between all subtribes of investigated Cichorieae (8 groups, 58 characters), *Prenanthes purpurea* as a member of the subtribe Hypochaeridinae→100% discrimination
5	Discrimination possibility between all subtribes of investigated Cichorieae (8 groups, 58 characters), *Prenanthes purpurea* as a member of the subtribe Lactucinae)→100% discrimination
6	Discrimination possibility between all subtribes of investigated Cardueae (4 groups, 69 characters), →98.6% discrimination (*Onopordum acanthium*)
7	Discrimination possibility between all investigated genera (except *Helminthotheca echioides*, 32 groups, 83 characters→100% discrimination

## Supplementary Materials

Supplementary information is available online at http://www.mdpi.com/2218-0532/84/4/585/s1. Figure S1: 50% majority rule consensus tree of three subtribes and 13 genera from the Cardueae; Figure S2: Ancestral character reconstructions based on the 50% majority rule consensus from Figure 2; Table S3: Summary of anatomical character states observed in 58 species of the Cardueae and Cichorieae.
