# Supplementary material for: Suitability of Root and Rhizome Anatomy for Taxonomic Classification and Reconstruction of Phylogenetic Relationships in the Tribes Cardueae and Cichorieae (Asteraceae)
Source: Sci Pharm. 2016 May 27;84(4):585–605. doi: 10.3390/scipharm84040585 (PMC5198019; doi:10.3390/scipharm84040585)
Supplement: Supplementary file 1 [file scipharm-84-00585-s001.pdf]

Supplementary Materials: Suitability of Root and Rhizome Anatomy for Taxonomic Classification and Reconstruction of Phylogenetic Relationships in the Tribes Cardueae and Cichorieae (Asteraceae)

Supplementary figure 1: 50% majority rule consensus tree of three subtribes and 13 genera from the Cardueae based on nrDNA single nucleotide polymorphisms and indel polymorphism. The tree was reconstructed from 7500 retained trees (out of 10,000; burnin = 2500) inferred using MrBayes. Posterior probabilities are given to the right of nodes. Numbers above branches are bootstrap values obtained for taxonomically equivalent clades running an independent maximum parsimony analysis on the same character set and accessions using PAUP (number of bootstrap replicates was 1000).

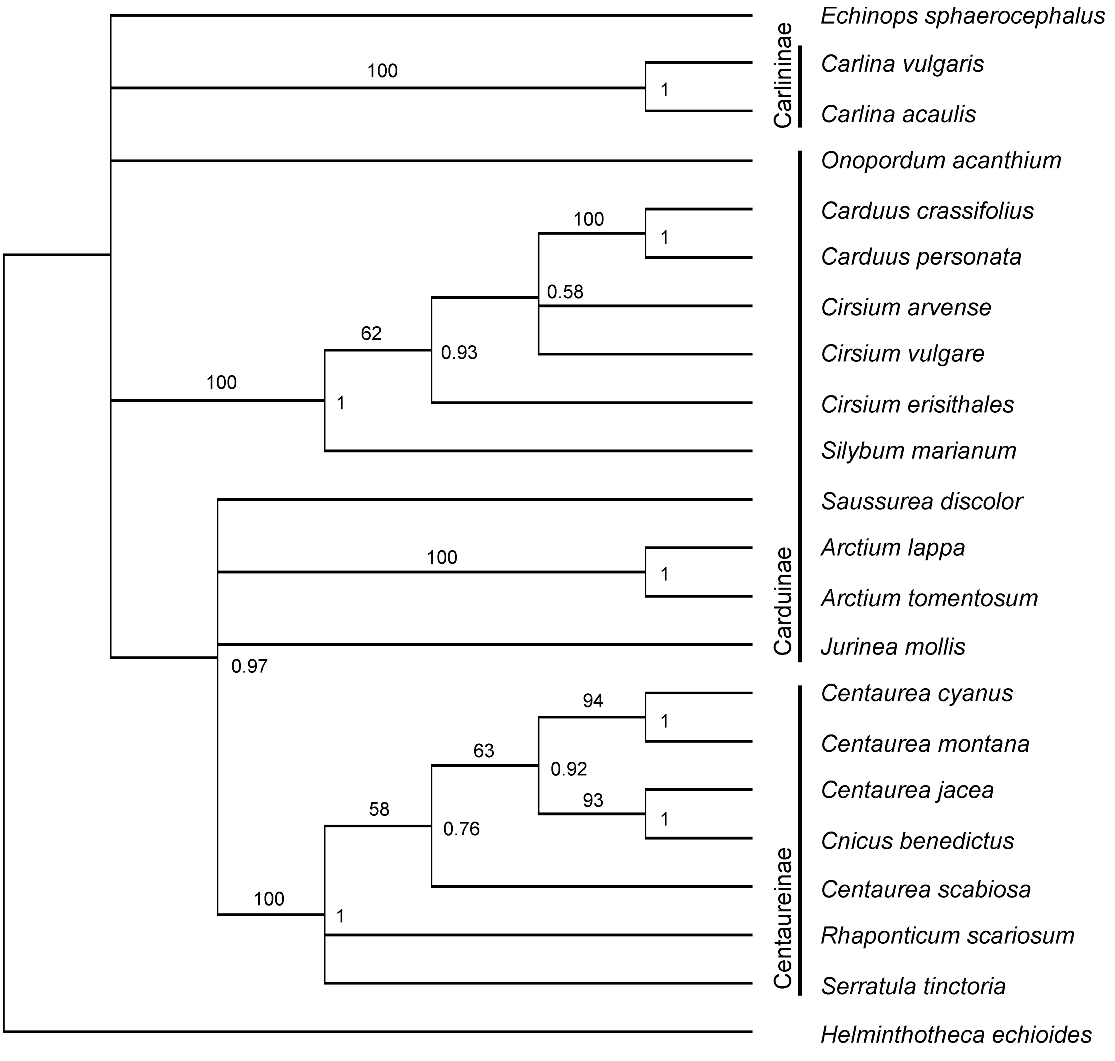

Supplementary figure 2: Ancestral character reconstructions based on the 50% majority rule consensus from Figure 2. The character states of ancestral nodes were inferred using Mesquite under the parsimony criterion. (a) cortex durability; (b) fibers in secondary xylem; (c) phellem; (d) phellem cells; (e) vessel perforation; (f) pits of vessels; (g) medullary rays; (h) vessel perforation; (i) vessel arrangement. Character states as indicated by the legend.

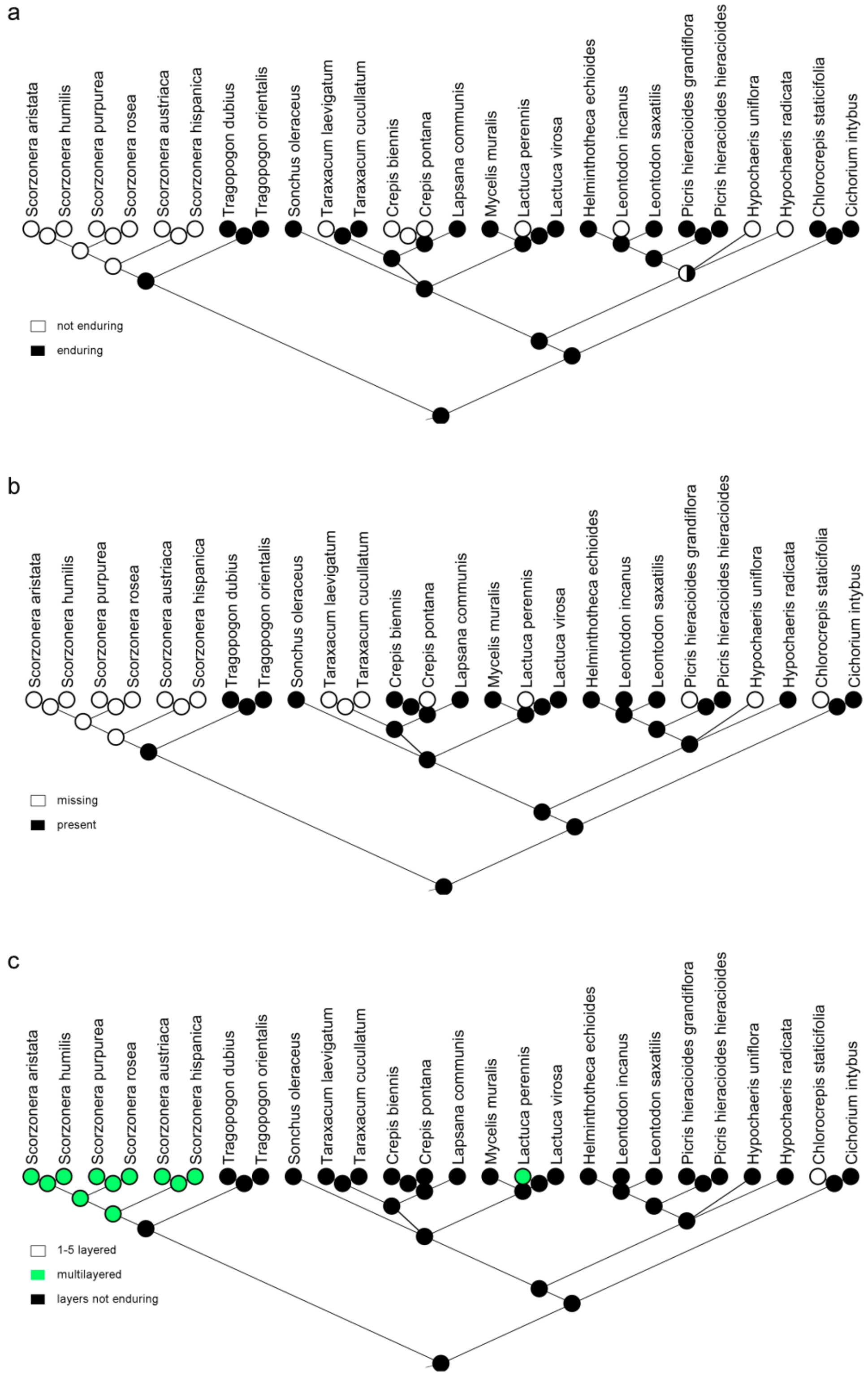

d

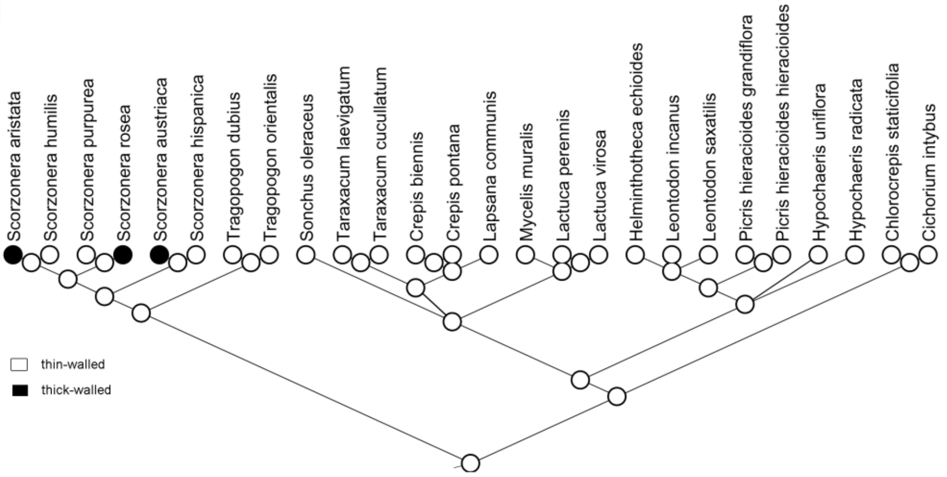
Scorzonera aristata
Scorzonera humilis
Scorzonera purpurea
Scorzonera rosea
Scorzonera austriaca
Scorzonera hispanica
Tragopogon dubius
Tragopogon orientalis
Sonchus oleraceus
Taraxacum laevigatum
Taraxacum cucullatum
Crepis biennis
Crepis pontana
Lapsana communis
Mycelis muralis
Lactuca perennis
Lactuca virosa
Helminthotheca echioides
Leontodon incanus
Leontodon saxatilis
Picris hieracioides grandiflora
Picris hieracioides hieracioides
Hypochaeris uniflora
Hypochaeris radicata
Chlorocrepis staticifolia
Cichorium intybus
thin-walled
thick-walled

e

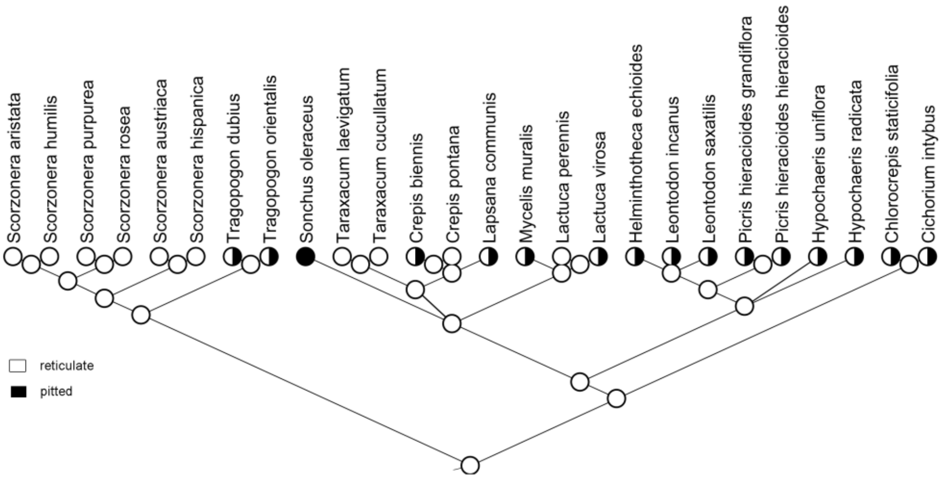
Scorzonera aristata
Scorzonera humilis
Scorzonera purpurea
Scorzonera rosea
Scorzonera austriaca
Scorzonera hispanica
Tragopogon dubius
Tragopogon orientalis
Sonchus oleraceus
Taraxacum laevigatum
Taraxacum cucullatum
Crepis biennis
Crepis pontana
Lapsana communis
Mycelis muralis
Lactuca perennis
Lactuca virosa
Helminthotheca echioides
Leontodon incanus
Leontodon saxatilis
Picris hieracioides grandiflora
Picris hieracioides hieracioides
Hypochaeris uniflora
Hypochaeris radicata
Chlorocrepis staticifolia
Cichorium intybus
reticulate
pitted

f

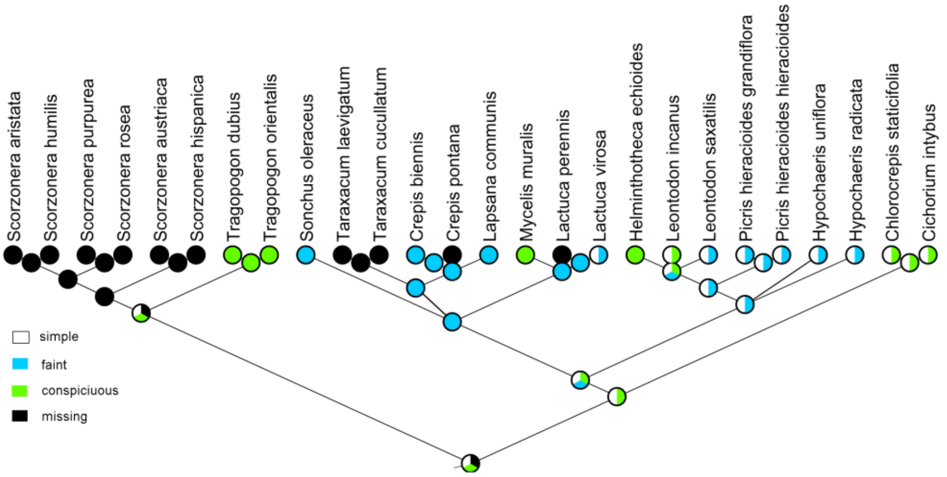
Scorzonera aristata
Scorzonera humilis
Scorzonera purpurea
Scorzonera rosea
Scorzonera austriaca
Scorzonera hispanica
Tragopogon dubius
Tragopogon orientalis
Sonchus oleraceus
Taraxacum laevigatum
Taraxacum cucullatum
Crepis biennis
Crepis pontana
Lapsana communis
Mycelis muralis
Lactuca perennis
Lactuca virosa
Helminthotheca echioides
Leontodon incanus
Leontodon saxatilis
Picris hieracioides grandiflora
Picris hieracioides hieracioides
Hypochaeris uniflora
Hypochaeris radicata
Chlorocrepis staticifolia
Cichorium intybus
simple
faint
conspiciuous
missing

g

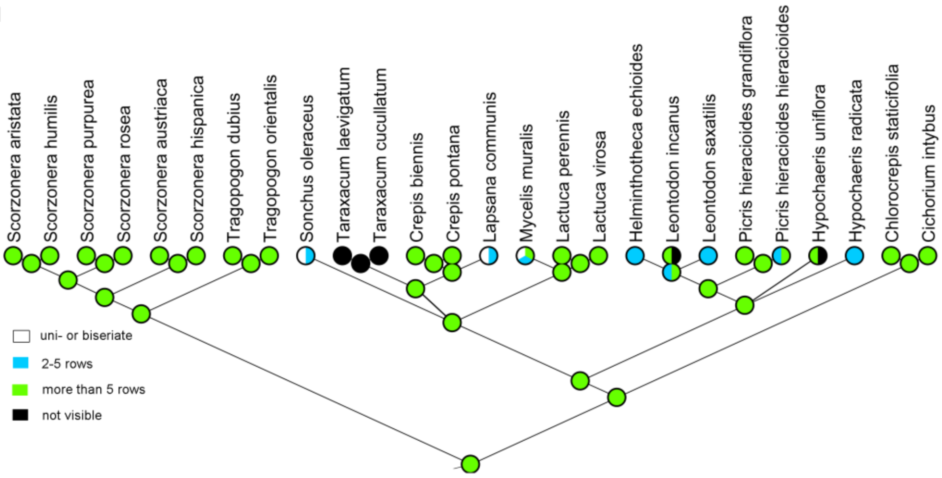
Scorzonera aristata
Scorzonera humilis
Scorzonera purpurea
Scorzonera rosea
Scorzonera austriaca
Scorzonera hispanica
Tragopogon dubius
Tragopogon orientalis
Sonchus oleraceus
Taraxacum laevigatum
Taraxacum cucullatum
Crepis biennis
Crepis pontana
Lapsana communis
Mycelis muralis
Lactuca perennis
Lactuca virosa
Helminthotheca echioides
Leontodon incanus
Leontodon saxatilis
Picris hieracioides grandiflora
Picris hieracioides hieracioides
Hypochaeris uniflora
Hypochaeris radicata
Chlorocrepis staticifolia
Cichorium intybus
uni- or biseriate
2-5 rows
more than 5 rows
not visible

h

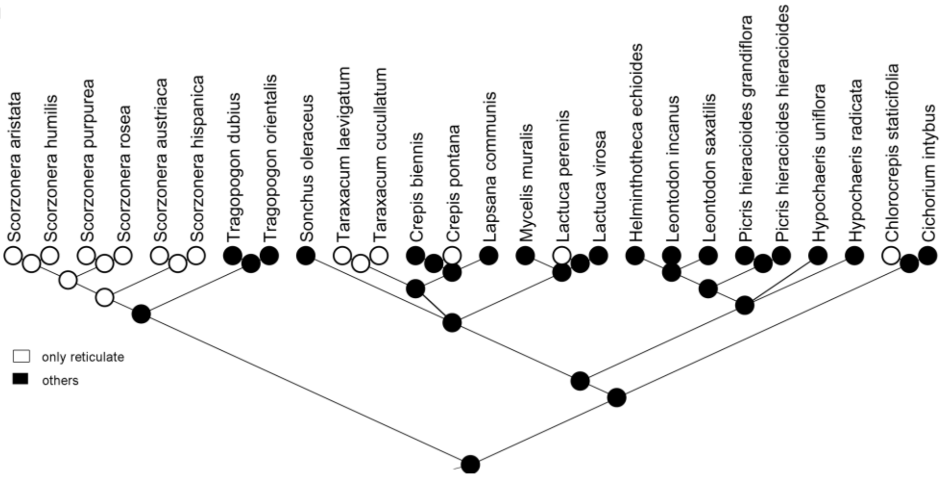
Scorzonera aristata
Scorzonera humilis
Scorzonera purpurea
Scorzonera rosea
Scorzonera austriaca
Scorzonera hispanica
Tragopogon dubius
Tragopogon orientalis
Sonchus oleraceus
Taraxacum laevigatum
Taraxacum cucullatum
Crepis biennis
Crepis pontana
Lapsana communis
Mycelis muralis
Lactuca perennis
Lactuca virosa
Helminthotheca echioides
Leontodon incanus
Leontodon saxatilis
Picris hieracioides grandiflora
Picris hieracioides hieracioides
Hypochaeris uniflora
Hypochaeris radicata
Chlorocrepis staticifolia
Cichorium intybus
only reticulate
others

i

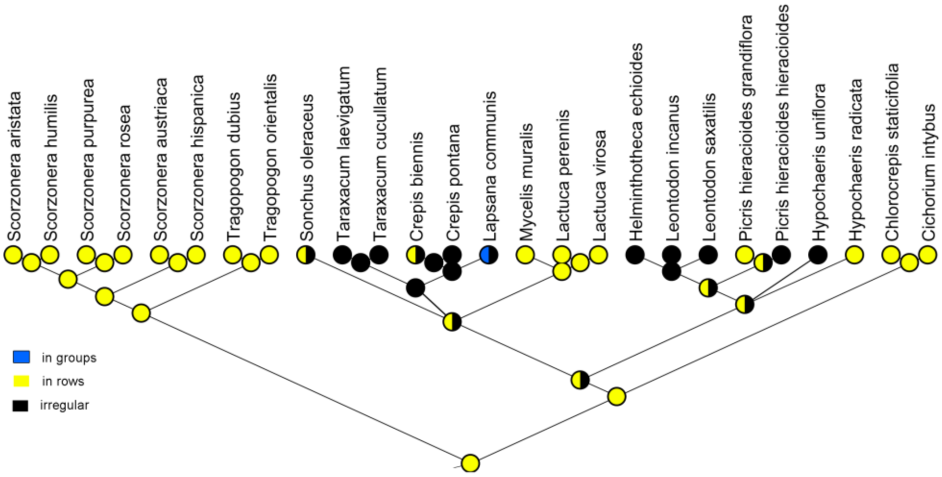
Scorzonera aristata
Scorzonera humilis
Scorzonera purpurea
Scorzonera rosea
Scorzonera austriaca
Scorzonera hispanica
Tragopogon dubius
Tragopogon orientalis
Sonchus oleraceus
Taraxacum laevigatum
Taraxacum cucullatum
Crepis biennis
Crepis pontana
Lapsana communis
Mycelis muralis
Lactuca perennis
Lactuca virosa
Helminthotheca echioides
Leontodon incanus
Leontodon saxatilis
Picris hieracioides grandiflora
Picris hieracioides hieracioides
Hypochaeris uniflora
Hypochaeris radicata
Chlorocrepis staticifolia
Cichorium intybus
in groups
in rows
irregular

Supplementary table 3: Summary of anatomical character states observed in 58 species of the Cardueae and Cichorieae: See Table 2 for definition of states. Superscripts provide links to further reading about the anatomy of underground parts of respective species: numbers see references.

Taxon	Subtribe	1. Subterranean Organ	2. Tissue Dominating In Extension	3. Cortex Durability	4. Endodermis	5. Fibers in Secondary Xylem	6. Fibers in Secondary Phloem	7. Sclereids	8. Endodermal Resin Ducts	9. SG1	10. SG2	11. SG3	12. SG4	13. Secretory Cavities (1 = Missing)	14. Arrangement of Laticifers	15. Phellem	16. Phellem Cells	17. Vessel Perforation	18. Pits of Vessels	19. Medullary Rays	20. Pith Cells	21. Arrangement OF Vessels	22. Crystal Needles
**Cardueae**																							
Arctium lappa [7,8]	Carduinae	3	1	1	1	2	2	1	2	1	1	1	1	1	1	3	1	1 2	1 2	3	1	4	1
Arctium tomentosum [7,8]	Carduinae	3	1	1	1	2	2	1	2	1	1	1	1	1	1	3	1	1 2	1 2	3	1	4	1
Carduus crassifolius [7,8]	Carduinae	3	1	2	1	2	2	2 3	2	1	1	1	1	1	1	3	1	1 2	2	3	1	1	1
Carduus personata [7,8]	Carduinae	1	1	2	1	2	2	2 3 4	2	1	1	1	1	1	1	3	1	1 2	3	3	6	5	1
Cirsium arvense [7,8]	Carduinae	1 2 3	1	2	1	2	2	2 3	2	1	5	1	1	1	1	3	1	2	3	2 3	1 2 3	5	1
Cirsium erisithales [7,8]	Carduinae	1 2	1	1	1	2	2	1	2	1	1	1	1	1	1	3	1	1 2	3	3	2	1	1
Cirsium vulgare [7,8]	Carduinae	3	1	2	2	2	2	2 3	2	1	1	1	1	1	1	3	1	1 2	1 2 3	3	1	5	1
Jurinea mollis [8]	Carduinae	3	1 2	2	2	2	2	1	2	1	1	1	1	1	1	3	1	1 2	1 2	3	1	4	1
Onopordum acanthium [7,8,25]	Carduinae	3	1	2	2	2	2	1	2	1	1	1	1	1	1	3	1	2	3	2 3	1	5	1
Saussurea discolour [34]	Carduinae	5	1 2	1	1	2	1	1	1	1	2 3	1	1	1	1	2	1	1 2	1	4	1	5	1
Saussurea pygmaea [34]	Carduinae	5	1 2	1	1	1	1	1	1	1	2 3	1	1	1	1	2	1	1	-	4	1	5	1
Silybum marianum [7,8,25]	Carduinae	3	1	2	1	2	2	1	2	1	1	1	1	1	1	3	1	2	3	2 3	1	2 4	1
Carlina acaulis [7,8,25]	Carlininae	3	1 2	1	1	2	2	1	2	3 5	1	1	1	1	1	3	1	2	1 3	2 3	1	3 5	2
Carlina vulgaris [7,8,25]	Carlininae	3	1	2	2	2	1	1	2	1	2 3	1	1	1	1	3	1	2	3	1	1	5	1
Centaurea jacea [7,8]	Centaureinae	1 4	1 2	2	2	2	2	2 3	2	2 3	1	1	3	1	1	3	1	1 2	1 2	1 3	3	5	1
Centaurea scabiosa [7,8]	Centaureinae	3	1 2	1	1	2	2	2 3	2	2	2	2	1	1	1	3	1	1 2	3	3	1	1	1
Cnicus benedictus [7,8]	Centaureinae	3	1	2	2	2	2	1	2	1	1	1	1	1	1	3	1	2	3	3	1	5	1
Centaurea montana [8]	Centaureinae	4	1 3	2	2	2	1	1	2	2 3	1	1	2	1	1	3	1	1 2	2	3	2	4 5	1
Centaurea cyanus [7,8]	Centaureinae	3	1	2	2	2	1	1	2	1	2	1	1	1	1	3	1	1 2	3	1 2	1	1 5	1
Rhaponticum scariosum [7,8]	Centaureinae	1 2 3	1 2	2	1	2	2	1	2	1	2 4 5	1	1	1	1	3	1	1	-	3	1 2	5	1
Serratula tinctoria [8]	Centaureinae	4	3	2	2	2	1	1	2	1	1	1	1	1	1	-	-	2	1 2	4	1	2	1
Echinops sphaerocephalus [8,25]	Echinopsidinae	1 2 3 4	1 2	2	1	2	2	3	2	1	1	1	1	2	1	3	1	1 2	3	2 3	1 2	1 5	1
**Cichorieae**																							
Chondrilla juncea	Chondrillinae	1	1	1	1	2	1	1	1	1	1	1	1	1	4	2	1	2	2	1 3	6	5	1
Willemetia stipitata	Chondrillinae	1	3	2	2	2	1	1	1	1	1	1	1	1	4	3	1	1 2	2 3	2 3	2	5	1
Chlorocrepis staticifolia	Cichoriinae	3	1 2	2	2	1	1	1	1	1	1	1	1	1	4	1	1	1 2	1 3	3	1	4	1
Cichorium intybus [9,25]	Cichoriinae	3	1 2	2	1	2	1	1	1	1	1	1	1	1	3	3	1	1 2	1 3	3	1	3	1

Crepis aurea	Crepidinae	1	3	2	2	1	1	4 5 6	1	1	1	1	1	1	4	3	1	1 2	2	2 3	3 4	5	1
Crepis biennis	Crepidinae	4	1 2	1	1	2	1	1	1	1	1	1	1	1	2	3	1	1 2	2	3	1	3 5	1
Crepis pontana [9]	Crepidinae	3	1 2	1	1	1	1	1	1	1	1	1	1	1	2	3	1	1	-	3	1	5	1
Lapsana communis [25]	Crepidinae	3	1	2	1	2	1	1	1	1	1	1	1	1	4	3	1	1 2	2	1 2	1	2 5	1
Taraxacum cucullatum [6,9]	Crepidinae	3	2	2	1	1	1	1	1	1	1	1	1	1	2	3	1	1	-	4	1	5	1
Taraxacum laevigatum [9]	Crepidinae	3	2	1	1	1	1	1	1	1	1	1	1	1	2	3	1	1	-	4	1	5	1
Hieracium murorum	Hieraciinae	1	3	2	2	2	1	1	1	1	1	1	1	1	4	3	1	1 2	1 2	3	2	5	1
Hieracium pilosella	Hieraciinae	1	3	2	1	2	2	1	1	1	1	1	1	1	4	3	1	1 2	1 2	2 3	2	5	1
Aposeris foetida [6,9]	Hyoseridinae	1	2	2	1	1	1	1	1	1	1	1	1	1	4	3	1	1 2	1	3	5	5	1
Sonchus oleraceus	Hyoseridinae	3	1 2	2	2	2	1	1	1	1	1	1	1	1	3 4	3	1	2	2	1 2	1	3 5	1
Helminthotheca echioides	Hypochaeridinae	4	1 2	2	2	2	1	1	1	1	1	1	1	1	3	3	1	1 2	3	2	1	5	1
Hypochaeris radicata [6,9]	Hypochaeridinae	4	2	1	1	2	1	1	1	1	1	1	1	1	2 3	3	1	1 2	1 2	2	1	3 4	1
Hypochaeris uniflora [6,9]	Hypochaeridinae	1 2 3	2	1	1	1	1	1	1	1	1	1	1	1	2	3	1	1 2	1 2	3 4	1 2	5	1
Leontodon hispidus [6,9]	Hypochaeridinae	1	3	2	2	2	1	1	1	1	1	1	1	1	4	3	1	1 2	1 2	3	2 3	5	1
Leontodon incanus [6,9]	Hypochaeridinae	3	2	1	1	2	1	1	1	1	1	1	1	1	2	3	1	1 2	1 3	3 4	1	5	1
Leontodon saxatilis	Hypochaeridinae	4	3	2	2	2	1	1	1	1	1	1	1	1	2	3	1	1 2	1 2	2	1	5	1
Picris hieracioides subsp. Grandiflora	Hypochaeridinae	4	1 2	2	2	1	1	1	1	1	1	1	1	1	2	3	1	1 2	1 2	3	1	3	1
Picris hieracioides subsp. Hieracioides	Hypochaeridinae	4	1	2	1	2	2	2 3 4 5 6	1	1	1	1	1	1	4	3	1	1 2	1 2	2 3	1	5	1
Prenanthes purpurea	Hypochaeridinae	1	1	2	2	2	1	1	1	1	1	1	1	1	4	2	1	2	3	3	2	5	1
Scorzoneroides autumnalis [6,9]	Hypochaeridinae	1	3	2	1	1	1	1	1	1	1	1	1	1	4	3	1	1 2	1 2	3	2	4	1
Scorzoneroides helvetica [6,9]	Hypochaeridinae	1	3	2	1	2	1	1	1	1	1	1	1	1	4	3	1	1 2	1 2	3	2	4	1
Cicerbita alpina	Lactucinae	1	1 2	2	2	2	1	1	1	1	1	1	1	1	4	1	1	2	3	3	2	5	1
Mycelis muralis	Lactucinae	1 3 4	1	2	2	2	1	1	1	1	1	1	1	1	4	3	1	1 2	3	1 2 3	1 2	3	1
Lactuca perennis	Lactucinae	3	1 2	1	1	1	1	1	1	1	1	1	1	1	3	2	1	1	-	3	1	4	1
Lactuca virosa	Lactucinae	3	1 2	2	1	2	1	1	1	1	1	1	1	1	3 4	3	1	1 2	1 2	3	1	3	1
Scorzonera aristata [9]	Scorzonerinae	1 2 3	1 2	1	1	1	1	1	1	1	1	1	1	1	3	2	2	1	-	3	1 2	4	1
Scorzonera austriaca [9]	Scorzonerinae	6	4	1	1	1	1	1	1	1	1	1	1	1	3	2	2	1	-	3	1	3	1
Scorzonera hispanica [9]	Scorzonerinae	1 2 3	1 2	1	1	1	1	1	1	1	1	1	1	1	3	2	1	1	-	3	2	3	1
Scorzonera humilis [9]	Scorzonerinae	1 2 3	1 2	1	1	1	1	5	1	1	1	1	1	1	3	2	1	1	-	3	2	3 4	1
Scorzonera purpurea [9]	Scorzonerinae	3	1 2	1	1	1	1	1	1	1	1	1	1	1	3	2	1	1	-	3	1	3	1
Scorzonera rosea [9]	Scorzonerinae	3	1 2	1	2	1	1	1	1	1	1	1	1	1	3	2	2	1	-	3	1	3 4	1
Tragopogon dubius [9]	Scorzonerinae	3	1 2	2	2	2	1	1	1	1	1	1	1	1	3	3	1	1 2	3	3	1	3	1
Tragopogon orientalis [9]	Scorzonerinae	3	1 2	2	2	2	1	1	1	1	1	1	1	1	3	3	1	1 2	3	3	1	3	1
